# Retraction: Regulatory B Cell Function Is Suppressed by Smoking and Obesity in *H*. *pylori*-Infected Subjects and Is Correlated with Elevated Risk of Gastric Cancer

**DOI:** 10.1371/journal.pone.0265376

**Published:** 2022-03-09

**Authors:** The PLOS ONE Editors

The *PLOS ONE* Editors have been unable to verify the identities, affiliations, contributions and contact email addresses for several of the authors listed on this article [1].

In response to follow-up queries, corresponding authors Zhendong Zheng and Ji J. Yuan stated that there was an error in the original listed affiliations; specifically, Roswell Park Cancer Institute, Georgetown University Medical Center, University of Maryland Medical Center, and University Hospital, University of British Columbia were incorrectly included. Revised affiliation information was provided, but the *PLOS ONE* Editors were unable to verify the updated affiliations.

In light of the above concerns, the *PLOS ONE* Editors retract this article.

JJY did not agree with retraction. ZZ did not respond directly to the retraction decision. GL, HW, TBL, DZS, ZS, SZ, PAP, MLT, GMG, PTM, RSD, AJJ and YMR could not be reached.
